# Construction and application of star polycation nanocarrier-based microRNA delivery system in *Arabidopsis* and maize

**DOI:** 10.1186/s12951-022-01443-4

**Published:** 2022-05-07

**Authors:** Jia Yang, Shuo Yan, Shipeng Xie, Meizhen Yin, Jie Shen, Zhaohu Li, Yuyi Zhou, Liusheng Duan

**Affiliations:** 1 State Key Laboratory of Plant Physiology and Biochemistry, Engineering Research Center of Plant Growth Regulator, Ministry of Education & College of Agronomy and Biotechnology, China Agricultural University, No. 2 Yuanmingyuan West Road, Haidian District, Beijing, 100193 People’s Republic of China; 2 Department of Plant Biosecurity and MARA Key Laboratory for Monitoring and Green Management, China Agricultural University, No. 2 Yuanmingyuan West Road, Haidian District, Beijing, 100193 People’s Republic of China; 3 State Key Laboratory of Chemical Resource Engineering, Beijing Laboratory of Biomedical Materials, Beijing University of Chemical Technology, No. 15 North Third Ring East Road, Chaoyang District, Beijing, 100029 People’s Republic of China; 4 College of Plant Science and Technology, Beijing University of Agriculture, Beijing, 102206 People’s Republic of China

**Keywords:** Gene silencing, MiRNA delivery, Nanoparticle, Plant biotechnology, Star polycation

## Abstract

**Background:**

MicroRNA (miRNA) plays vital roles in the regulation of both plant architecture and stress resistance through cleavage or translation inhibition of the target messenger RNAs (mRNAs). However, miRNA-induced gene silencing remains a major challenge in vivo due to the low delivery efficiency and instability of miRNA, thus an efficient and simple method is urgently needed for miRNA transformation. Previous researches have constructed a star polycation (SPc)-mediated transdermal double-stranded RNA (dsRNA) delivery system, achieving efficient dsRNA delivery and gene silencing in insect pests.

**Results:**

Here, we tested SPc-based platform for direct delivery of double-stranded precursor miRNA (ds-*MIRNA*) into protoplasts and plants. The results showed that SPc could assemble with ds-*MIRNA* through electrostatic interaction to form nano-sized ds-*MIRNA*/SPc complex. The complex could penetrate the root cortex and be systematically transported through the vascular tissue in seedlings of *Arabidopsis* and maize. Meanwhile, the complex could up-regulate the expression of endocytosis-related genes in both protoplasts and plants to promote the cellular uptake. Furthermore, the SPc-delivered ds-*MIRNA* could efficiently increase mature miRNA amount to suppress the target gene expression, and the similar phenotypes of *Arabidopsis* and maize were observed compared to the transgenic plants overexpressing miRNA.

**Conclusion:**

To our knowledge, we report the first construction and application of star polycation nanocarrier-based platform for miRNA delivery in plants, which explores a new enable approach of plant biotechnology with efficient transformation for agricultural application.

**Graphical Abstract:**

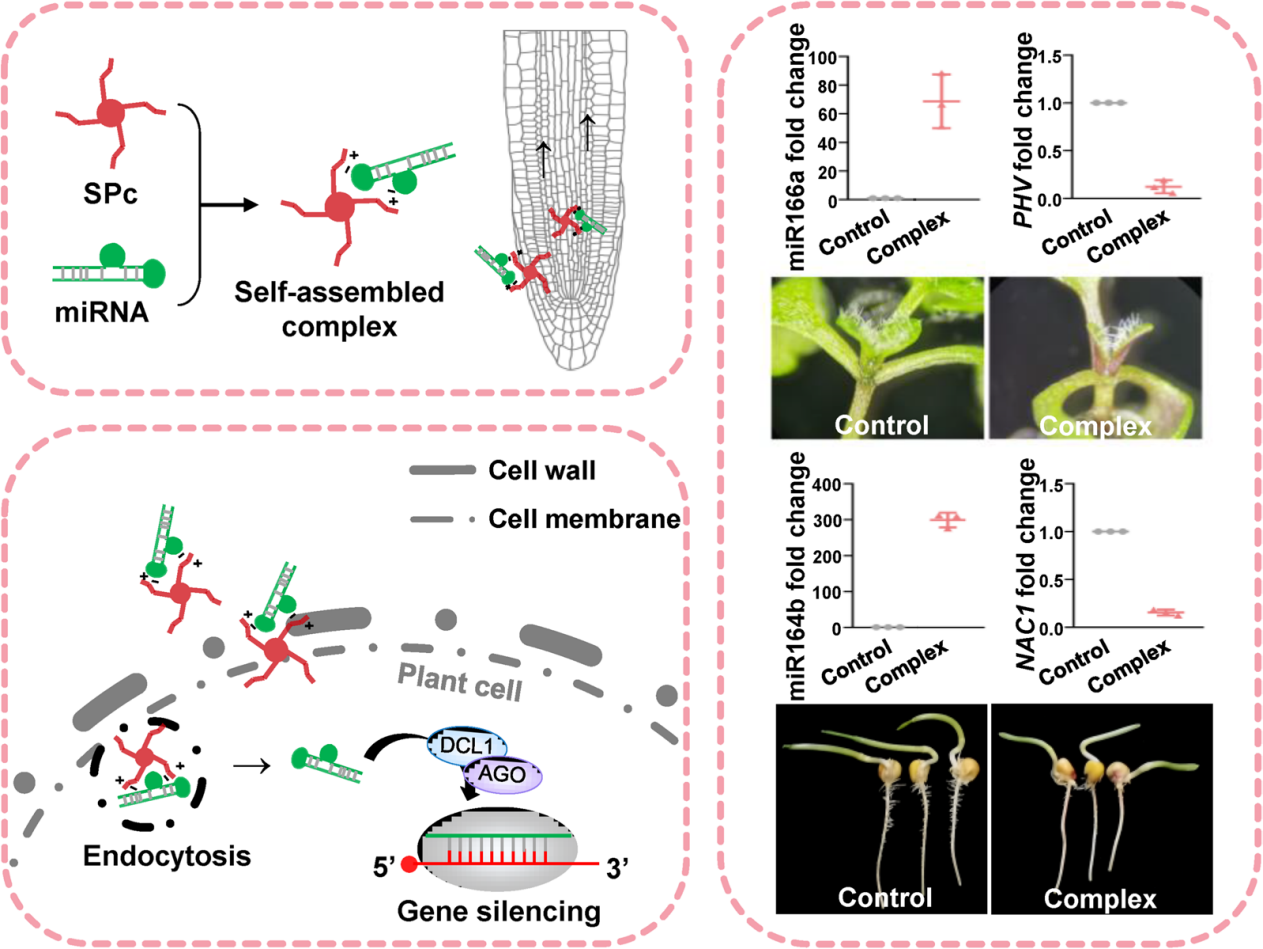

**Supplementary Information:**

The online version contains supplementary material available at 10.1186/s12951-022-01443-4.

## Introduction

Accumulating evidences have suggested that microRNAs (miRNAs), a class of small regulatory RNAs that direct the enzymolysis of mRNA molecules, are linked to a variety of biological processes, including plant development, protein degradation, environmental stress response and pathogen invasion [1–3]. The biogenesis of plant miRNAs involves the synthesis of double-stranded precursor miRNA (ds-*MIRNA*) that is cleaved by DICER-LIKE proteins and condensed into 20–24 nt double-stranded miRNA [4]. The cleaved miRNA duplexes are then associated with ARGONAUTE proteins to recognize the target messenger RNAs (mRNAs), thereby degrading the target mRNA to further inhibit the protein expression [2]. Therefore, efficient miRNA delivery has great potential to regulate plant architecture and stress resistance in agricultural production. However, the miRNA application is constrained by the absent of efficient miRNA delivery method. The upper limit of particle size for penetrating the plant cell wall is below 20 nm. Furthermore, the repulsion of miRNAs to the negatively-charged cell membrane and instability of miRNAs in vivo also contribute to the low gene silencing efficiency [5–7]. These critical hurdles related to miRNA delivery should be overcome for functional gene identification [8]. Consequently, *Agrobacterium*-mediated and biolistic methods have been widely explored as miRNA vectors, but each with considerable limitations [9]. Nanoparticles are emerging as delivery vehicles for biomolecules in interdisciplinary research of biotechnology and nanotechnology [10]. While nanoparticle-mediated RNA delivery has been extensively explored in biological detection, disease therapy and pest control, its great potential application in plant systems is still needed exploration [11–15]. Previous studies have successfully applied nanoparticles to deliver plasmid DNA [16–18], dsRNA [19] or siRNA [20–22] to intact plant cells, showing that the nanoparticles can reach the plant tissues, cells and subcellular locations that are previously inaccessible. These representative nanoparticles include dendrimer [19], clay nanosheet [20], carbon nanotube [18, 21], gold nanoclusters [22], carbon dot [23], etc. Nano-delivery systems have exploited the high degree of control over morphology and surface functionalized groups, the diverse conjugation chemistries available for cargo conjugation, and the permeability through plant tissues to overcome the biological barriers [24]. These advancements in nanotechnology have suggested a great potential for the safe and highly efficient delivery of biomolecules such as miRNA, CRISPR-Cas, and RNAi into plant cells, and it is expected to be a promising method for overcoming the limitations of miRNA delivery [25].

Star polycation (SPc) nanoparticle has been designed and synthesized to construct a SPc-based transdermal dsRNA delivery system, which can be applied for efficient gene silencing and effective control toward aphids [26, 27]. Subsequent application of SPc is limited to drug/dsRNA delivery for controlling plant diseases and pests [28–33]. Thus, the important function of SPc has not been fully elucidated, and the SPc may be applied to construct a miRNA delivery platform for plants. Herein, we tested the interaction between ds-*MIRNA* and SPc through agarose gel electrophoresis, determined the particle size of ds-*MIRNA*/SPc complex through dynamic light scattering (DLS), and observed the morphology characterization of ds-*MIRNA*/SPc complex through transmission electron microscopy (TEM) to illustrate the effective combination between ds-*MIRNA* and SPc. Then, we traced the SPc-delivered miRNA in protoplasts and plant roots, and examined the expression of important genes related with clathrin-mediated endocytosis to illustrate the enhanced delivery of SPc-loaded miRNA in vivo and in vitro. Finally, the *Arabidopsis* miR166 and maize miR164 were tested through SPc-mediated miRNA delivery system, and the obtained phenotypes were consistent to those of transgenic plants. To our knowledge, it is the first attempt to construct and apply nanocarrier-based miRNA delivery system for plant gene silencing, which explores a new enable approach of plant biotechnology with efficient transformation for agricultural application.

## Methods

### Synthesis of SPc and ds-*MIRNA*

**Scheme 1:**
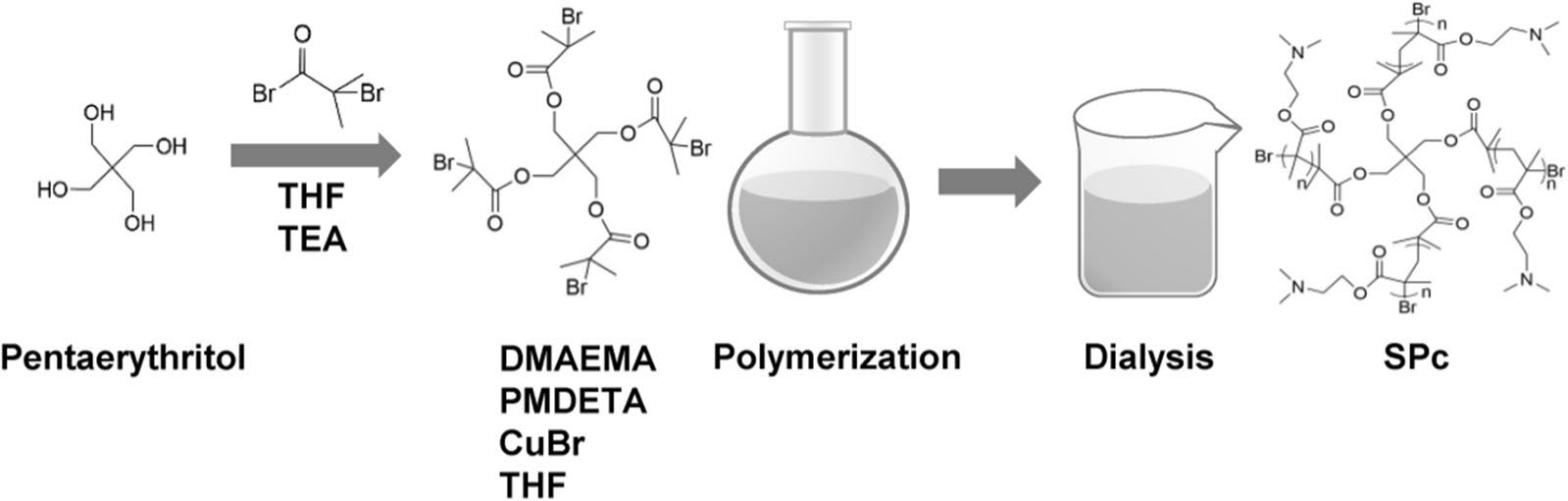
Synthesis route of Pt-Br and SPc

The SPc was synthesized according to the method described by Li et al. [27]. The star initiator Pt-Br was synthesized using pentaerythritol, and the polymerization of star initiator with DMAEMA was then carried out. The crude polymer was purified by dialysis, and the product of SPc was finally obtained as white powder and dissolved in ddH_2_O (Scheme 1). The structure of SPc was confirmed by ^1^H nuclear magnetic resonance (^1^H NMR), and the molecular weight was analyzed by gel permeation chromatography (GPC) [27, 34]. MiR166a targets five members of the HD-ZIP III transcription factor genes, such as *PHAVOLUTA* (*PHV*), *PHABULOSA* (*PHB*), *REVOLUTA* (*REV*), *ATHB8* and *ATHB15/CORONA*, leading to severe shoot apical meristem (SAM) damage and even lethal to *Arabidopsis* [35–38]. MiR164b directs *NAC1* mRNA cleavage in vivo and inhibits the lateral root development in maize [39]. Two miRNAs were taken as examples to test the nanocarrier-based miRNA delivery system. The fragments of Ath-*MIR166a* (170 bp, Araport: AT2G46685) and Zma-*MIR164b* (127 bp, miRBase: MI0001471) were cloned into the pCUN(m)-GFP (Takara, China) and transformed into the *Escherichia coli* DH5α strain (Invitrogen, USA). The plasmid was extracted and verified by Sanger sequencing (Tsingke, China). The ds-*MIRNA* fragments were prepared at the concentration of 1 μg/μL in nuclease-free water using T7 RiboMAX expression RNAi system (Promega, USA).

### Loading capacity measurement and preparation of the ds-*MIRNA*/SPc complex

The Zma-*MIR164b*/SPc complexes were prepared at the mass ratios of 5:1, 3:1, 1:1 and 1:3 (ds-*MIRNA*: SPc), respectively to examine their binding ability. Each mixture (ds-*MIRNA*: 3 μg) was mixed and incubated for 15 min at room temperature before 1% agarose gel electrophoresis. To get higher efficiency, ds-*MIRNA* should be added into SPc solution [40]. Then the SPc could assemble with ds-*MIRNA* spontaneously into ds-*MIRNA*/SPc complex in aqueous solution. 20 μL of Zma-*MIR164b*/SPc complex was dipped on the pH test strips (SSS Reagent, China) to determine the pH value. The pH values of ddH_2_O, Zma-*MIR164b* and SPc were also tested.

### Particle size and zeta potential measurement

The particle size and zeta potential value of Zma-*MIR164b*/SPc complex at the mass ratio of 1:1 (ds-*MIRNA* concentration: 1 μg/μL) were tested in triplicate at 25 °C with a Zetasizer Nano ZS (Malvern Instruments, USA).

### Complex morphology characterization

The 20 μL solutions of Zma-*MIR164b*/SPc complexes at the mass ratios of 1:1, 1:2 and 1:3 (ds-*MIRNA* concentration: 1 μg/μL) were dropped onto the 200-mesh carbon-formvar film coated grids (Zhong Xing Bairui Technology Co. Ltd., China), and aired-dried before observation using transmission electron microscope (TEM, JSM-7500F, Japan). The particle sizes of Zma-*MIR164b*/SPc complexes were measured using ImageJ 1.8 (National Institutes of Health, USA).

### Plant materials and growth conditions

The transgenic *Arabidopsis* strains with WOX5::GFP provided by Prof. J. Zhang (China Agricultural University, China) were used for fluorescent tracing of miRNA. The *Arabidopsis* strains (Col) were used for RNAi efficiency determination of miRNA. The transgenic *Arabidopsis* strain overexpressing Ath-miR166a in the Columbia background (Col) was generated by the floral dip method [41], which was applied for observing the phenotype of Ath-miR166-induced gene silencing. Three *Arabidopsis* strains were cultured at a 16 h light and 8 h dark under 22 °C and 60% humidity condition.

The maize strain (ZD958) was used for fluorescent tracing, RNAi efficiency determination and endocytosis test of miRNA. Transgenic maize strain overexpressing Zma-miR164e was constructed based on the maize (ND101) background by Center for Crop Functional Genomics and Molecular Breeding of China Agricultural University, which was used for observing the phenotype of Zma-miR164-induced gene silencing. Two maize strains were cultured under the growth conditions of 25 °C day temperature and 18 °C night temperature, accompanied by 16 h light and 8 h dark.

Protoplasts were prepared according to the method described by Zelazny et al. [42] for fluorescent tracing and endocytosis test of miRNA. After incubation under the dark conditions for 14 days, the mesophyll protoplasts were isolated from etiolated leaves of maize strain (ZD958) digested in enzyme solution. The protoplasts were collected after the centrifugation and washing in W5 solution (154 mM NaCl, 125 mM CaCl_2_, 5 mM KCl and 4 mM MES at pH 5.7). The filtered solution was centrifuged, and the pelleted protoplasts were resuspended in washing solution to prepare the protoplasts with desired concentration (3.5 × 10^6^ protoplast/mL).

### Penetration of SPc-loaded ds-*MIRNA* into plant roots

The 1% volume of surfactant Alkyl Polyglucoside (Wanhua, China) was added into the ds-*MIR166a*/SPc complex (mass ratio of 1:1), and the surfactant can reduce the surface tension of hydrophilic nanocomplex and help the complex adhere to plant roots [30, 43]. The ds-*MIR166a* was labeled with CXR Reference dye (Promega, USA) as a fluorescent probe, and the ds-*MIR166a*/SPc complex (1.5 μg ds-*MIR166a*) was then applied to the root tip of WOX5::GFP *Arabidopsis* seedling. The ddH_2_O and naked ds-*MIR166a* were applied as controls. The fluorescent intensities of GFP and CXR dye were recorded and calculated at 6 and 12 h after the treatment using ImageJ 1.8 (National Institutes of Health) from 3 independent samples.

The maize (ZD958) with uniform taproot length was selected, and its 3 mm taproot-tip was excised and soaked in the 5 μL solution of ds-*MIR164b*/SPc complex (mass ratio of 1:1, and 2.5 μg ds-*MIR164b* per plant). The ddH_2_O and naked ds-*MIR164b* were applied as controls. The fluorescent signal was observed using fluorescence microscopy (Zesis 710 meta, Germany), and the fluorescent intensity was calculated at 12 h after the treatment using ImageJ 1.8 (National Institutes of Health) from 3 independent samples.

### Penetration of SPc-loaded ds-*MIRNA* into protoplasts and the endocytosis examination

The fluorescent ds-*MIR164b* delivered by SPc (5.5 μg ds-*MIR166a* per 1 mL protoplast) was added to the fresh protoplasts of maize. The ddH_2_O and naked ds-*MIR164b* were applied as controls. The fluorescent photos were taken after 2 h incubation. The treated protoplasts were collected for RNA extraction using the Total RNA Kit (Magen, China), and then reverse-transcribed into cDNA using TRUEscript 1st Strand cDNA synthesis Kit (Aidlab Biotechnologies, China). The expression of endocytosis-related genes such as *CHC* (GRMZM2G073792), *SAL1* (GRMZM2G117935), *Rab* (GRMZM2G075719), *AP2* (GRMZM2G092741), *EHD1* (GRMZM2G052740) and *ARF* (GRMZM2G083546) was determined using quantitative real-time PCR (qRT-PCR), which was performed with Step One Plus Real-Time PCR system (Applied Biosystems, USA) using Power SYBR^®^ Green Master Mix (Applied Biosystems). The parameters were: pre-denaturation at 95 °C for 30 s, followed by 40 cycles of denaturation at 95 °C for 5 s, annealing at 60 °C for 34 s, and a melting curve ramp was constructed to confirm that no reaction produced the nonspecific amplification. The housekeeping genes *AtActin-2* (AT3G18780) for *Arabidopsis* and *ZmUBC* (GRMZM2G419891) for maize were used as the reference genes. Primers were listed in Additional file 1: Table S1. QRT-PCR data were analyzed using the 2^−∆∆CT^ method described by Livak and Schmittgen [44]. For each sample, qRT-PCR was carried out triplicate technical repeated independently from the same isolated RNA batch to obtain the mean values. Triplicate biological replicates were used for each treatment.

### RNAi efficiency of SPc-delivered ds-*MIRNA* in *Arabidopsis* and maize

For RNAi efficiency test in *Arabidopsis*, the ds-*MIR166a*/SPc complex (1.5 μg ds-*MIR166a*) was applied to the root surface of *Arabidopsis* (Col) every 24 h, and RNAi efficiency was determined on 6 days after the treatment. The ddH_2_O and naked ds-*MIR166a* were applied as controls. Total RNA was extracted from *Arabidopsis*, and reverse-transcribed into cDNA. The ds-*MIR166a* amount was determined using qRT-PCR similarly as above from 3 independent samples. The stem-loop qRT-PCR was performed using 3 independent samples to quantify the Ath-miR166a amount according to the method described by Sun et al. [45]. The expression of target genes such as *PHV* (AT1G30490), *PHB* (AT2G34710), *REV* (AT5G60690), *ATHB8* (AT4G32880) and *ATHB15* (AT1G52150) was determined using qRT-PCR from 3 independent samples. For phenotype record, *Arabidopsis* growth parameters were observed on 6 days after the treatment. Two new leaves (#5 and 6 leaf) were captured with an Olympus SEX16 confocal microscope (Olympus Corp., Japan), and the leaf area was analyzed using ImageJ 1.8 from 4 seedlings. The leaves of 10-day-old transgenic *Arabidopsis* strain overexpressing Ath-miR166a was also observed.

For RNAi efficiency test in maize, the ds-*MIR164b*/SPc complex (1.5 μg ds-*MIR164b*) was applied to the root surface of maize (ZD958) every 24 h, and RNAi efficiency was determined on 4 days after the treatment. The ddH_2_O and naked ds-*MIR164b* were applied as controls. The amounts of ds-*MIR164b* and Zma-miR164b were determined using qRT-PCR from 3 independent samples similarly. The expression of target gene *ZmNAC1* (GRMZM2G063522) was determined using qRT-PCR from 3 independent samples. Furthermore, the expression of above endocytosis-related genes was also determined using quantitative real-time PCR (qRT-PCR). For phenotype record, taproot length was measured. The number of lateral roots (LRs) was counted, and LR density was obtained using the formula of LR density = LR number/taproot length. Five day old of transgenic maize overexpressing miR164e was also used to record the taproot length, LR number and LR density. Each seedling was used to collect the data, which was repeated 15 times for maize (ZD958) and 13 times for transgenic maize.

### Statistical analysis

All data were analyzed using SPSS statistics 22.0 (SPSS Inc., USA), and the figures were prepared using GraphPad Prism 8.0. (GraphPad Software, http://www.graphpad.com). Significance was analyzed using one-way analysis of variance (ANOVA) with Duncan’s multiple range test or independent* t* test. *P* values over 0.05 were considered non-significant differences. The descriptive statistics were given as the mean value and standard deviations of the mean.

## Results and discussion

### Loading capacity of SPc and complex characterization

**Fig. 1:**
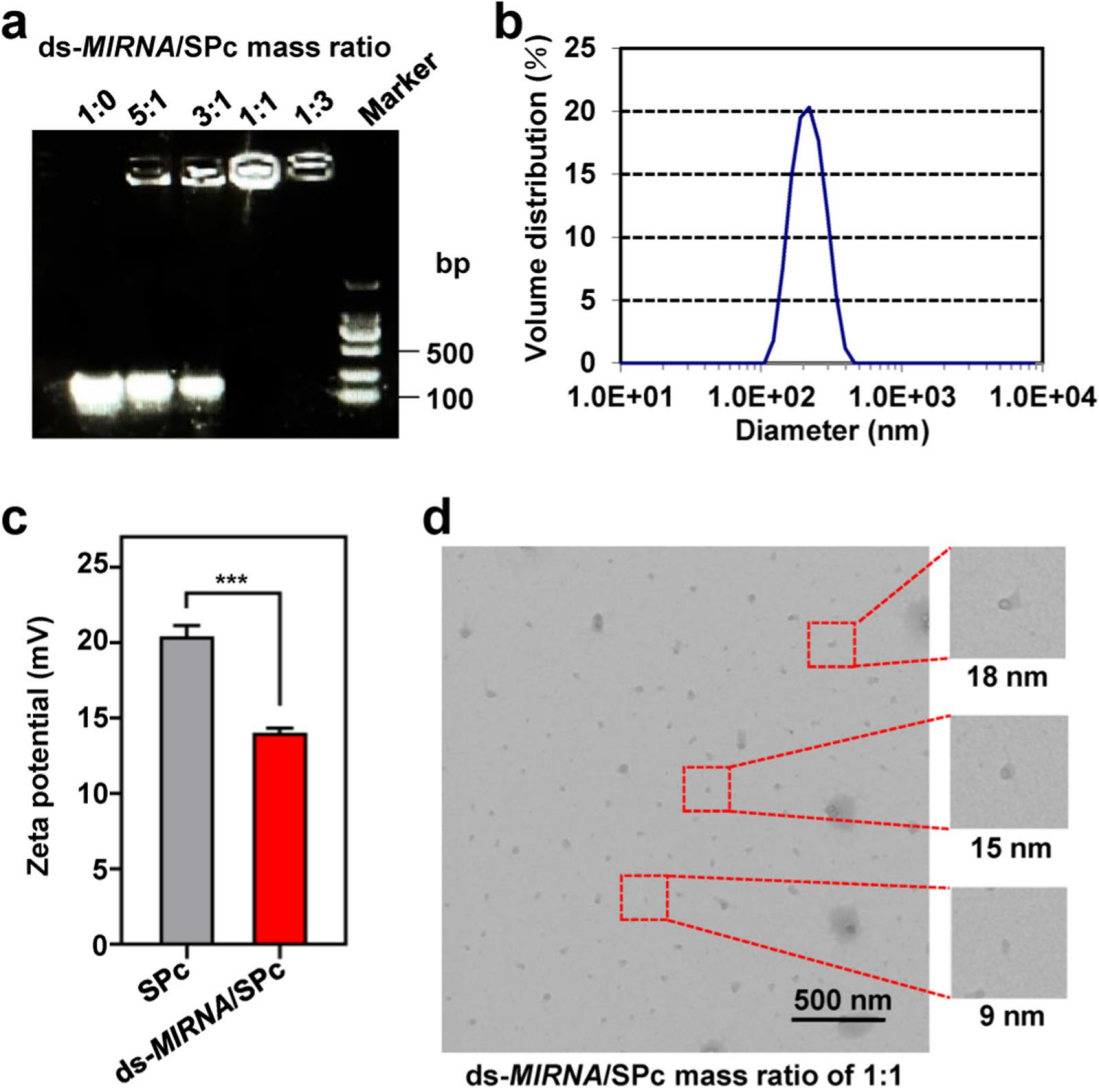
Self-assembly and characterization of ds-*MIRNA*/SPc complex. **a** Gel electrophoresis assay of ds-*MIR164b* retardation by SPc. The ds-*MIR164b*/SPc complexes (ds-*MIRNA*: 3 μg) were prepared at the mass ratios of 5:1, 3:1, 1:1 and 1:3 (ds-*MIRNA*: SPc), respectively. **b** Size distribution measurement of ds-*MIR164b*/SPc complex at the mass ratio of 1:1. **c** Zeta potential of ds-*MIR164b*/SPc complex at the mass ratio of 1:1. The “***” indicates the significant difference according to independent *t*-test at *P* < 0.001. **d** TEM images of ds-*MIR164b*/SPc complex at the mass ratio of 1:1. Small complexes were enlarged

The SPc is consisted of a hydrophilic shell with positively-charged tertiary amine, and the particle size of SPc is 100.5 nm with zeta potential value of 20.9 mV [27]. In the current study, the best mass ratio of ds-*MIRNA* and SPc was firstly determined at various mass ratios. As shown in Fig. 1a, the band’s intensity of the migrated ds-*MIR164b* gradually decreased with the reducing mass ratios, and the best mass ratio was 1:1 without electrophoresis shift, indicating that the SPc had excellent performance in combining with ds-*MIRNA*. The best mass ratio of SPc with dsRNA or DNA is 1:1 [27], and the SPc is a universal nanocarrier for combining with nucleic acids through electrostatic interaction, resulting in the electronegativity loss of ds-*MIRNA*. The pH of the ds-*MIRNA*/SPc complexes was around 7 (Additional file 1: Fig. S1). After the complexation, fewer acting sites of nucleic acid are exposed, which may increase the stability of nucleic acid/SPc complex in RNase A [46]. Furthermore, the multifunctional SPc can also be complexed with eugenol, thiamethoxam or osthole through electrostatic interaction [29, 31, 33].

Based on the data of dynamic light scattering, the average particle size of self-assembled ds-*MIR164b*/SPc complex was 253.6 ± 5.0 nm (Fig. 1b), which was similar to that of DNA/SPc complex [27]. Meanwhile, this self-assembly reduced the zeta potential value to + 14.03 mV (Fig. 1c). The increased particle size and reduced zeta potential of ds-*MIR164b*/SPc complex compared to SPc suggested the potential electrostatic adhesion of ds-*MIRNA* to SPc’s surface. Furthermore, the TEM images revealed that ds-*MIR164b*/SPc complex was consisted of spherical nanoparticles with small particle size below 20 nm (Fig. 1d). The particle size of ds-*MIR164b*/SPc complex differed between DLS and TEM results. In the TEM result, sometimes only the “cores” of the ds-*MIRNA*/SPc complex can be seen due to high electron density, while larger aggregates and hydrodynamic interactions often make the particles appear larger in the DLS result. However, the complexes could also form successfully when the mass ratio (ds-*MIRNA*:SPc) was decreased to 1:2 or 1:3 (Additional file 1: Fig. S2).

### Enhanced delivery of SPc-loaded ds-*MIRNA* into plant roots and protoplasts

**Fig. 2:**
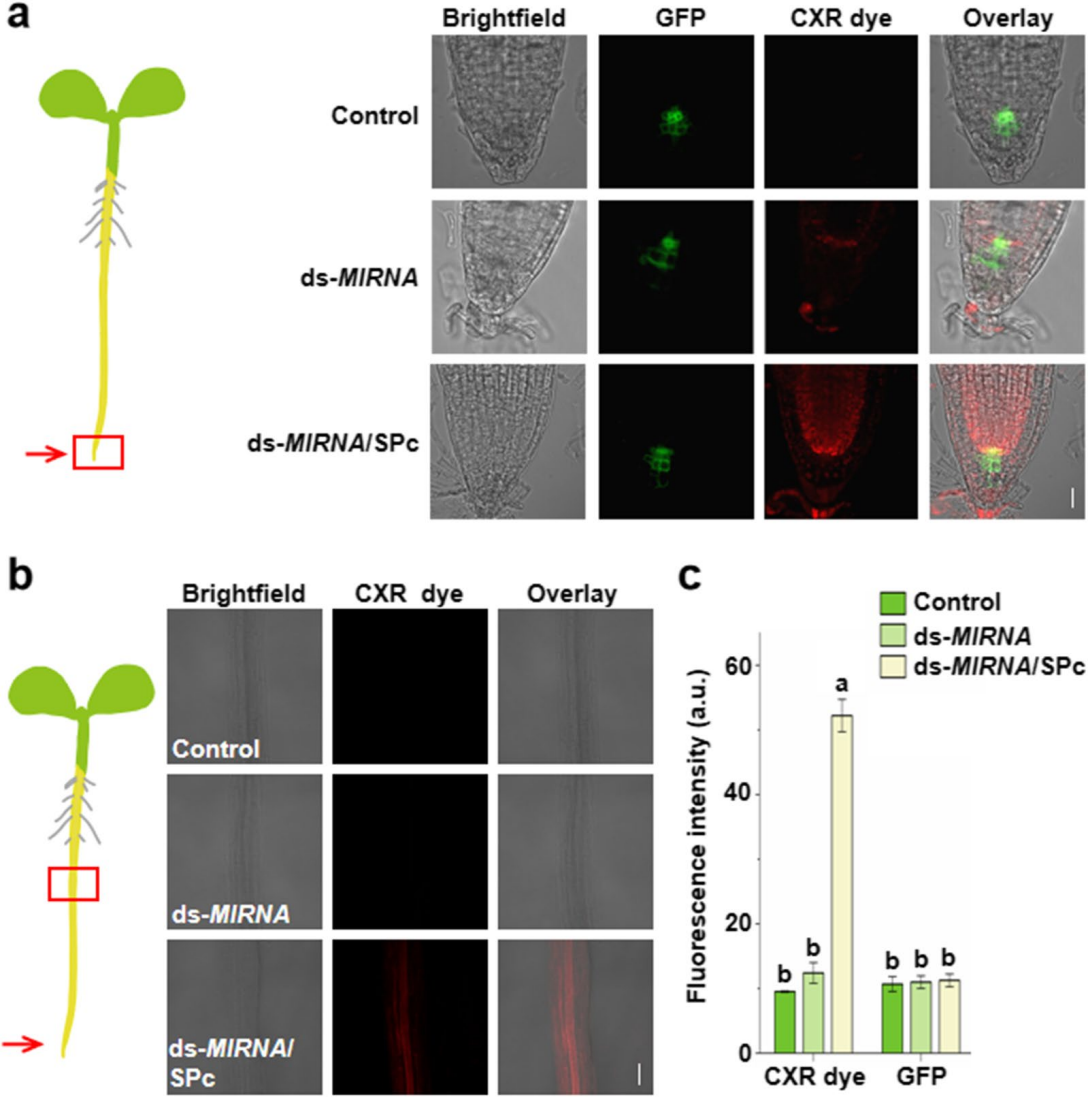
Enhanced delivery of SPc-loaded ds-*MIRNA* into *Arabidopsis* roots. **a** Schematic diagram of root application and representative confocal images of *Arabidopsis* roots at 6 after the treatment of various formulations. Red arrow and box indicate the application and observation site, respectively. Red: ds-*MIRNA*; Green: GFP; Scale bar: 20 µm. **b** Representative confocal images of *Arabidopsis* roots at 12 h after the treatment of various formulations. Red: ds-*MIRNA*; Scale bar: 50 µm. **c** Fluorescent quantitative analysis of *Arabidopsis* roots in **a**. Different letters on columns indicate significant differences (Duncan’s multiple range test, *P* < 0.05)

**Fig. 3:**
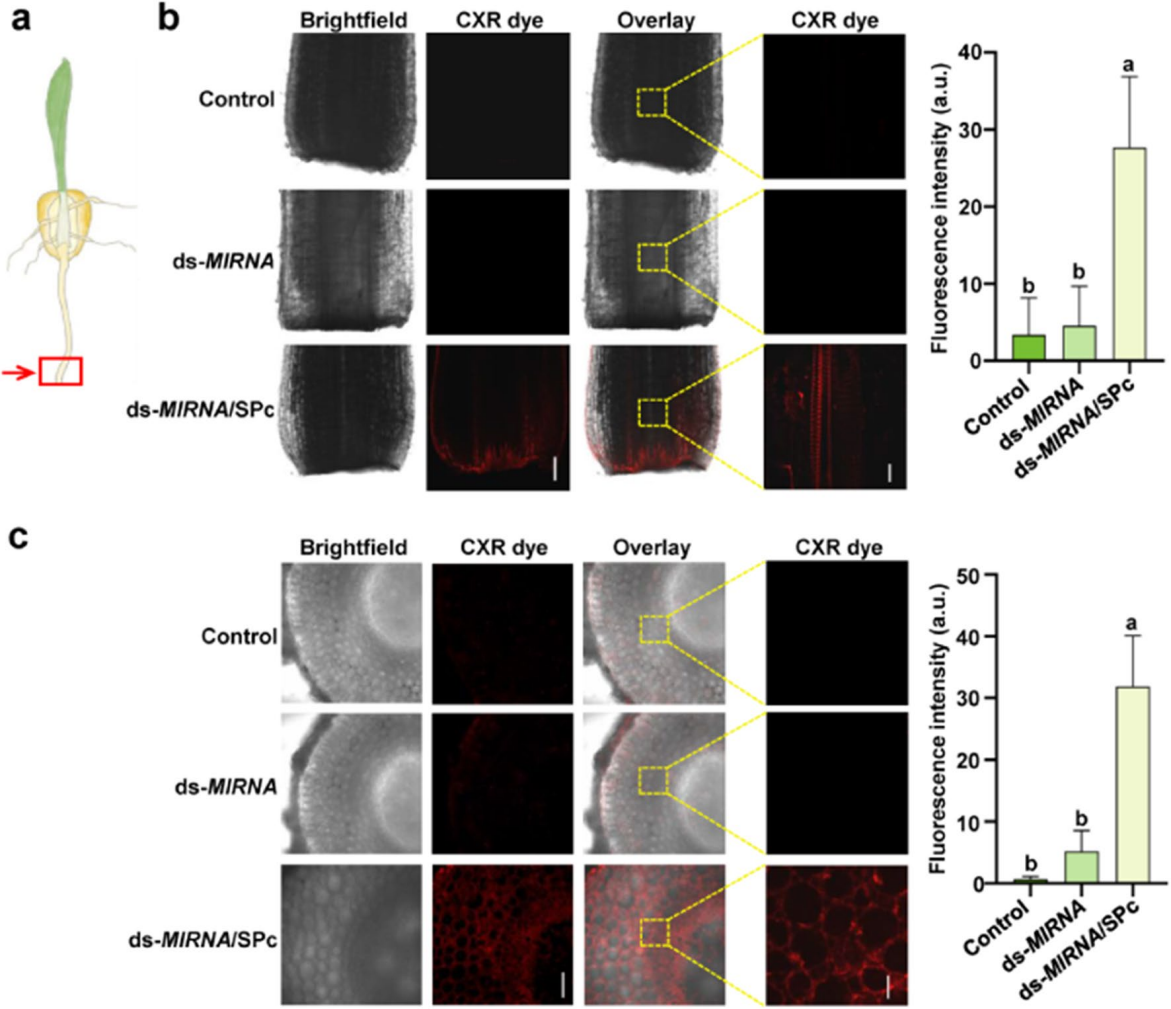
Enhanced delivery of SPc-loaded ds-*MIRNA* into maize roots. **a** Schematic diagram of root application. Red arrow and box indicate the application and observation site, respectively. **b** Representative longitudinal section images of roots and fluorescent analysis of SPc-delivered ds-*MIRNA* at 12 h after the treatment. Right and left scale bars indicate 200 and 20 µm, respectively. Different letters on columns indicate significant differences (Duncan’s multiple range test, *P* < 0.05). **c** Representative cross section images of roots and fluorescent analysis of SPc-delivered ds-*MIRNA* at 12 h after the treatment. Right and left scale bars indicate 200 and 100 µm, respectively

Root system is the most important organ for plants to absorb water and nutrients [47]. Previous study has confirmed that both positively and negatively-charged nanoparticles can be taken up and accumulated in *Arabidopsis* roots with the particle size less than 200 nm [48]. To this end, we firstly tested the SPc-delivered ds-*MIR166a* into the roots of *Arabidopsis* expressing the WOX5::GFP (Fig. 2). The WOX5 is specifically expressed in quiescent centre (QC), where locates in plant root meristem. The ds-*MIR166a*/SPc complex was applied to the root tip of *Arabidopsis*, and the different sections of roots were observed for tracing ds-*MIR166a*. As shown in Fig. 2a and c, the SPc-delivered ds-*MIR166a* could penetrate the root epidermis within 6 h, and the fluorescence was significantly stronger than naked ds-*MIR166a* (52.21 a.u. vs. 11.26 a.u.). As expected, control and naked ds-*MIR166a* treatments presented nearly no distinguishable fluorescence, indicating that naked ds-*MIRNA* could not penetrate into *Arabidopsis* root epidermis. Furthermore, the SPc-delivered ds-*MIR166a* could be tested in vascular tissues far away from the application site, which revealed that the SPc was able to transport ds-*MIR166a* upwards through vascular tissues (Fig. 2b). Meanwhile, the SPc could also promote the ds-*MIR164b* delivery into maize roots (Fig. 3). The fluorescent signal of ds-*MIR164b*/SPc complex could be easily examined in longitudinal section (Fig. 3b) and cross section (Fig. 3c) of maize roots, and that of naked ds-*MIR164b* was nearly undetectable. The current results indicated that the plant root could take up ds-*MIRNA* and transport it upwards via vascular route with the help of SPc.

To further confirm the enhanced plant-uptake of ds-*MIRNA*/SPc complex, we extracted intact protoplasts from maize leaves, and then checked their actual uptake of ds-*MIRNA*/SPc complexes. As shown in Additional file 1: Fig. S3, the fluorescent signal of SPc-delivered ds-*MIR166a* could be examined in the cytoplasm of protoplasts after 2 h incubation, whereas protoplasts incubated with naked ds-*MIR166a* showed nearly no detectable fluorescent signals. Thus, the SPc could promote the delivery of ds-*MIRNA* into both plant roots and protoplasts. Nanoparticles used for nucleic acid delivery in plants include mesoporous silica nanoparticle (MSNs), single walled carbon nanotubes (SWNTs), DNA-modified gold nanoparticles (AuNP), DNA nanostructures and layered double hydroxide nanosheets (LDH), and these nanoparticles are mainly used to deliver plasmid DNA, dsRNA and siRNA by leaf injection [24]. One publication has reported the efficient DNA/dsRNA delivery in *Arabidopsis* through the root application, and the dendrimer-delivered DNA/dsRNA can pass through the cell wall of the *Arabidopsis* root cap and root hair into root tissues [19]. Based on our previous studies, the SPc can assemble with dsRNA or botanical/synthetic pesticides to promote their plant uptake. For instance, the SPc-delivered dsRNA shows enhanced root uptake by soybean and radish seedlings [26, 30]. The uptake of SPc-loaded thiamethoxam, osthole and dinotefuran was increased to 1.69–1.84, 1.28 and 1.45–1.53 times compared with pesticide alone in radish seedlings, strawberry leaves and oilseed rapes, respectively [31–33].

### Enhanced endocytosis induced by SPc in protoplasts and maize

**Fig. 4:**
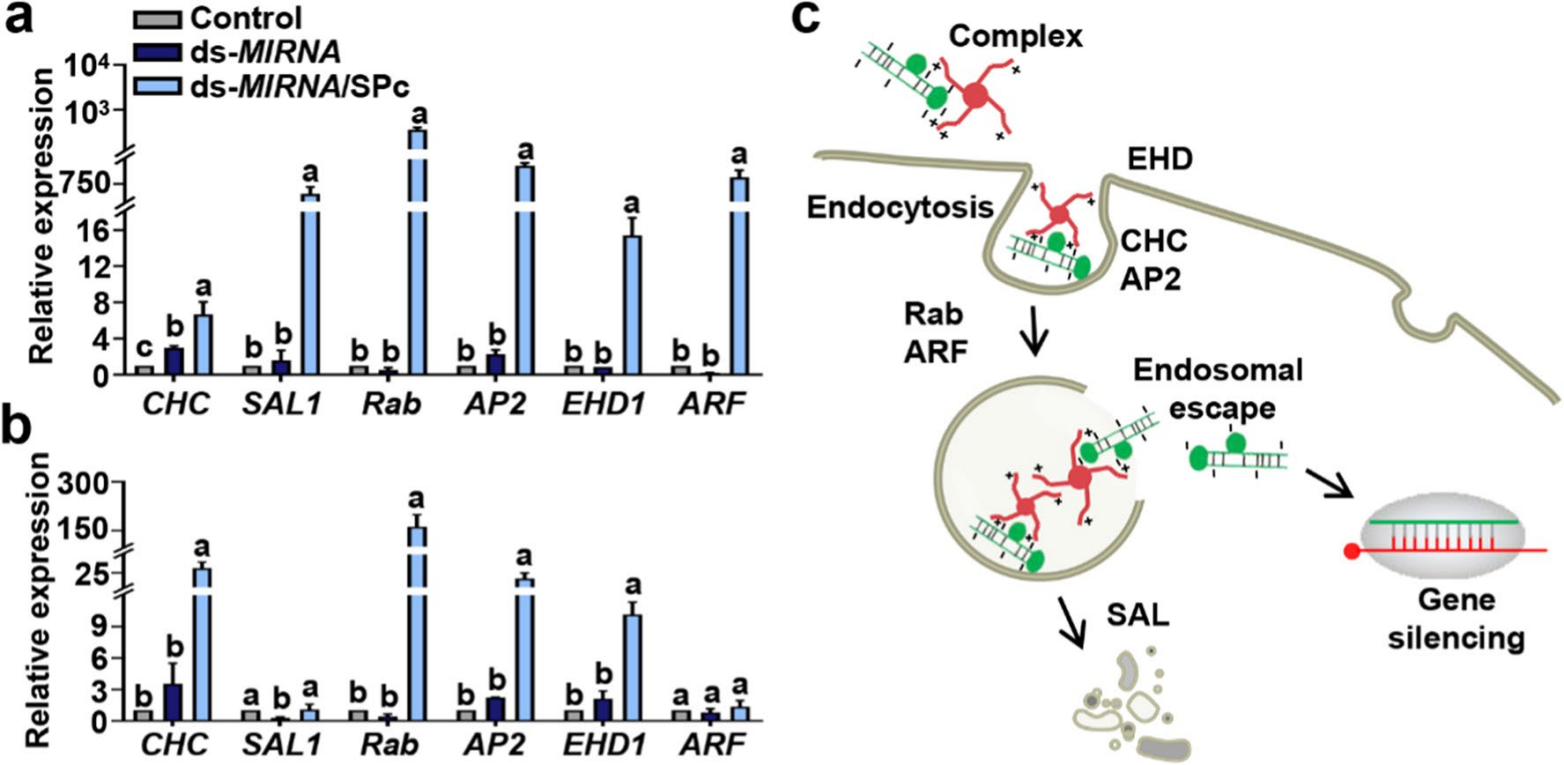
The SPc-delivered ds-*MIRNA* internalizes into protoplasts and maize through endocytosis. **a** Differentially expressed genes related to endocytosis in protoplasts. The treated protoplasts were collected after 2 h incubation. The housekeeping gene *ZmUBC* was used as the reference gene. Triplicate biological replicates were used for each treatment. Different letters on columns indicate significant differences among the treatments of control, ds-*MIRNA* and ds-*MIRNA*/SPc complex (Duncan’s multiple range test, *P* < 0.05). **b** Differentially expressed genes related to endocytosis in maize. The ds-*MIR164b*/SPc complex was applied to the root surface of maize every 24 h, and the expression of above endocytosis-related genes was determined on 4 days after the treatment. **c** Schematic illustration of the ds-*MIRNA*/SPc complex delivery into cells. The negatively-charged ds-*MIRNA* can be assembled with positively-charged SPc into ds-*MIRNA*/SPc complex, which is delivered into cells through endocytosis. The endocytosis-related genes were up-regulated, and the location of encoded proteins is shown

Endocytosis provides a major route for the entry of membrane proteins, lipids and extracellular molecules into the cells [49]. Based on our data, the SPc can up-regulate some key genes such as *Chc*, *AP2S1* and *Arf1* for activating clathrin-mediated endocytosis to improve the dsRNA delivery [46]. Furthermore, the SPc can also promote the plant uptake of chitosan through activating the endocytosis pathway [29]. To further explore the possible pathway for the plant-uptake of ds-*MIRNA*/SPc complex, we tested the expression of endocytosis-related genes in protoplasts and maize treated with ds-*MIRNA*/SPc complexes and naked ds-*MIRNA*. Compared to control and naked ds-*MIRNA*, ds-*MIRNA*/SPc complex remarkably up-regulated endocytosis-related genes in protoplasts (*CHC*, *SAL1*, *Rab*, *AP2*, *EHD1* and *ARF*) (Fig. 4a) and maize (*CHC*, *Rab*, *AP2* and *EHD1*) (Fig. 4b). However, the fold changes of gene expression generally varied more greatly in protoplasts than those in maize, and the expression of *ARF* and *SAL1* did not change significantly in maize, which was probably due to size screening of plant cell wall and later sampling time of maize.

The *CHC* gene encodes a major structural polypeptide of the surface lattice of clathrin-coated pits and vesicles, and the *AP2* encodes the sigma subunit of the Adaptor Protein 2 complex that drives endocytic vesicle formation at the plasma membrane [50–53]. ARF and Rab are members of the small G protein family. The *Rab* gene acts in various endocytic pathways, including internalization to early endosomes, transport to late endosomes and recycling of the plasma membrane [54]. The *ARF* gene regulates protein and lipid trafficking in eukaryotic cells through a regulated cycle of GTP binding and hydrolysis [55]. The up-regulation of these important genes revealed the activation of clathrin-mediated endocytosis of protoplasts and maize, and the schematic diagram was shown in Fig. 4c. Thus the enhanced endocytosis and small particle size for penetrating cell wall were the main mechanism of SPc-mediated improved ds-*MIRNA* delivery. After the cellular uptake, ds-*MIRNA*/SPc complex might be released from early endosome. The most popular accepted mechanism is “proton sponge” hypothesis, an escape by rupture of the endosome through osmotic swelling. It is based on the cationic polymers’ strong buffering capacity over a range of pH between 5 and 7, and the acidic environment in late endosome can promote the release of cationic polymers [46, 56, 57]. Next, some polyanions with a high affinity for cationic polymers can disassemble the nucleic acid/cationic polymer complex, which leads to the release of nucleic acid into cytoplasm [58]. On the other hand, some other nanoparticles can also be designed to possess functional properties such as stimulation responsiveness to aid nucleic acid release [14, 59].

### SPc-delivered ds-*MIRNA* shows efficient gene silencing effects on *Arabidopsis* and maize

**Fig. 5:**
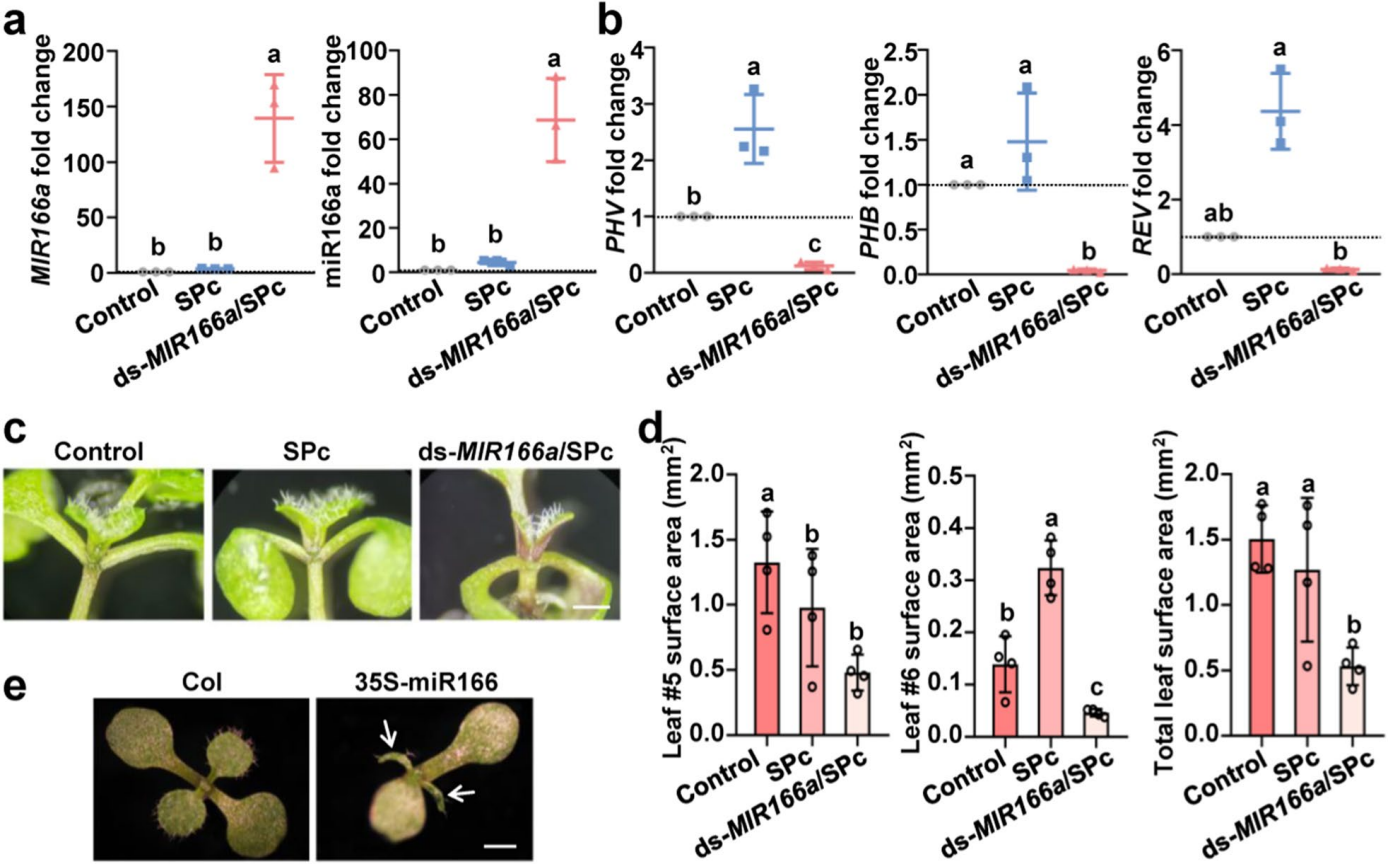
Ds-*MIRNA*/SPc complex-mediated gene silencing in *Arabidopsis*. **a** QRT-PCR assay for *MIR166a* and miR166a. The ds-*MIR166a*/SPc complex was applied to the root surface of *Arabidopsis* every 24 h, and the samples were collected on 6 days after the treatment. The housekeeping gene *AtActin-2* was used as the reference gene. Triplicate biological replicates were used for each treatment. Different letters on columns indicate significant differences (Duncan’s multiple range test, *P* < 0.05). **b** QRT-PCR assay for target genes. **c** Photos of *Arabidopsis* seedlings among various treatments. Scale bar: 500 μm. **d** Development evaluation of SAM by measuring the leaf # 5 and 6. **e** The photos of Col (wild type) and transgenic *Arabidopsis* strain overexpressing Ath-miR166a, which were taken with 10-day-old seedlings. Scale bar: 1 mm

As the ds-*MIRNA*/SPc complex could be efficiently transported into protoplasts and plant roots, we further explored whether the SPc-delivered ds-*MIRNA* could silence the target gene expression and induce the desired phenotypes in *Arabidopsis* and maize. For *Arabidopsis* test, the MiR166a was taken as an example, which targets five members of the HD-ZIP III transcription factor genes, and lead to severe impairs toward SAM in *Arabidopsis* [37]. As expected, the relative expressions of both *MIR166a* and miR166a were significantly increased by 139.35 and 68.64-fold, respectively after the treatment of ds-*MIR166a*/SPc complex (Fig. 5a). Furthermore, the expression of three target genes such as *PHV*, *PHV* and *REV* was strongly repressed by the application of ds-*MIR166a*/SPc complex, whereas the *ATHB8* and *ATHB15* expression was not repressed (Fig. 5b and Additional file 1: Fig. S4). The potential reason is probably that *PHV*, *PHB* and *REV* play key roles in two major processes during embryogenesis: the establishment of apical bilateral symmetry and SAM in *Arabidopsis*, whereas the *ATHB8* and *ATHB15* are mainly related to the development of vascular tissues [60].

The application of ds-*MIR166a*/SPc complex led to the impaired growth of *Arabidopsis* SAM compared to control (Fig. 5c). Regardless of the new leaf #5 or 6, the total leaf area of plants treated with ds-*MIR166a*/SPc complex was decreased by 62.25% than that of control (0.57 mm^2^ vs 1.51 mm^2^), exhibiting an apparent delay of SAM development (Fig. 5d), which was consistent with the phenotype of transgenic *Arabidopsis* strain overexpressing Ath-miR166a (Fig. 5e). Our result revealed that the SAM defect in *Arabidopsis* treated with ds-*MIR166a*/SPc complex was not as serious as 35S-miR166a overexpression material. The miR166 members have a unique spatiotemporal expression pattern in *Arabidopsis* [61], which influences the SAM development. The expression of 35S-miR166a in transgenic *Arabidopsis* strain was stronger than that of ds-*MIR166a* delivered by SPc, and the expression site was also important for inducing corresponding phenotype. Overall, the results indicated that the SPc-delivered ds-*MIR166a* could modulate SAM architecture by regulating the expression of target genes in *Arabidopsis*.

**Fig. 6:**
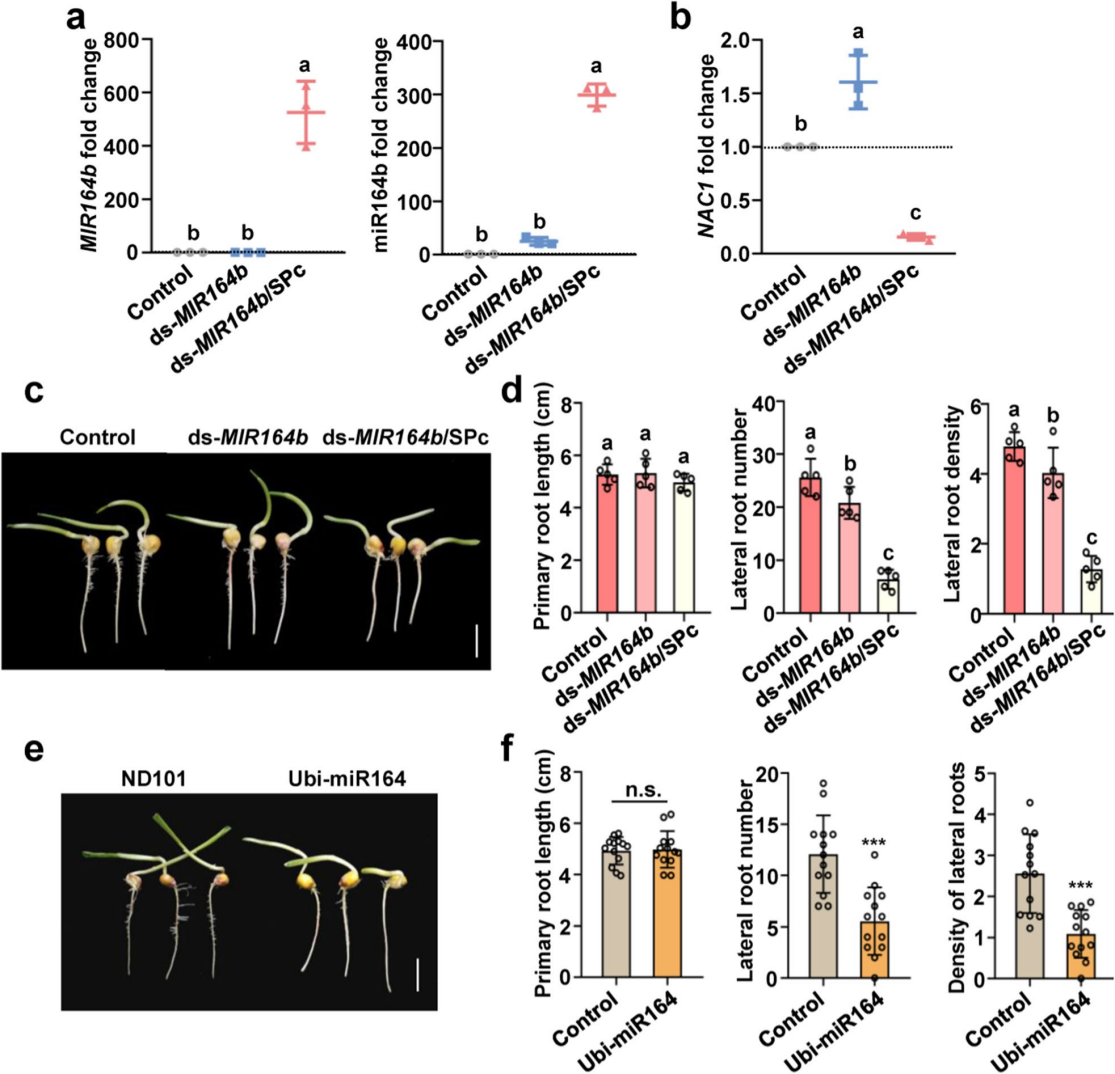
Ds-*MIRNA*/SPc complex-mediated gene silencing in maize. **a** QRT-PCR assay for *MIR164b* and miR164b. The ds-*MIR164b*/SPc complex was applied to the root surface of maize every 24 h, and the samples were collected on 4 days after the treatment. Triplicate biological replicates were used for each treatment. The housekeeping gene *ZmUBC* was used as the reference gene. Different letters on columns indicate significant differences (Duncan’s multiple range test, *P* < 0.05). **b** QRT-PCR assay for target gene *NAC1*. **c** Photos of maize among various treatments. Scale bar: 1 cm. **d** Growth evaluation of roots by measuring lateral root number and density. Fifteen seedlings were used to collect the data. **e** Photos of maize ND101 (widely type) and transgenic maize overexpressing miR164e. Scale bar: 1 cm. **f** Growth evaluation of roots by measuring lateral root number and density. Thirteen seedlings were used to collect the data. The “n.s.” means no significance, and the “***” indicates significant differences according to the independent t test (*P* < 0.001)

Previous studies have demonstrated that the miR164 directs *NAC1* mRNA cleavage in vivo at the 11th nucleotide of the complementary miR164 binding site, inhibiting the development of lateral roots [39]. Thus, the ds-*MIR164b* was also taken an example to examine the SPc-based gene silencing effects on the growth of maize roots. As shown in Fig. 6a, the SPc-delivered ds-*MIR164b* led to over 500 and 300-fold increase of *MIR164b* and miR164b amount compared to control and naked ds-*MIR164b*. Meanwhile, the down-regulation of target gene *NAC1* was also observed in the roots treated with ds-*MIR164b*/SPc complex (Fig. 6b). In addition, compared to control, LR number and LR density of maize treated with ds-*MIR164b*/SPc complex were significantly decreased (Fig. 6c, d and Additional file 1: Fig. S5), and the obtained phenotype was consistent to the transgenic maize overexpressing miR164e (Fig. 6e, f). These results further demonstrated that the SPc could deliver ds-*MIR164b* into plant cells to inhibit LR development in maize. Overall, our work established a star polycation-based platform for *MIRNA* delivery in plants. This approach may break through the limitations of leaf injection and provide a high efficient and convenient method for identification of functional genes and adjustment of agronomic traits. Furthermore, as the most important crop, maize has hard stalks and well-developed root systems, and this platform will be further used to defense against biotic and abiotic stresses in future.

## Conclusion

To sum up, we constructed an efficient SPc-based platform for direct delivery of ds-*MIRNA* into protoplasts and plants. The tertiary amines in the side chain of SPc could assemble with the negatively-charged ds-*MIRNA* at the best mass ratio of 1:1 through the electrostatic interaction. The formed nano-sized ds-*MIRNA*/SPc complex could penetrate the root cortex and be systematically transported through the vascular tissue in seedlings of *Arabidopsis* and maize. Meanwhile, the complex could up-regulate the expression of endocytosis-related genes such as *CHC*, *Rab*, *AP2*, etc. in both protoplasts and seedling roots to promote the cellular uptake. As expected, the SPc-delivered ds-*MIRNA* could efficiently increase mature miRNA amount to suppress the target gene expression in *Arabidopsis* and maize. The SPc-delivered ds-*MIR166a* and ds-*MIR164b* could inhibit the SAM architecture in *Arabidopsis* and lateral root development in maize, respectively, and the obtained phenotypes were consistent to corresponding transgenic plants. Our work is the first attempt to apply nanoparticle-based platform for miRNA delivery in plants, which explores a new enable approach of plant biotechnology with efficient transformation for agricultural application.

## Supplementary Information

**Additional file 1: Table S1.** The primers used in the current study. **Figure S1.** The pH test for ds-MIRNA/SPc complex on pH test strips. Different colors represent the corresponding pH values reference to the right color table. **Figure S2.** Representative TEM images of ds-*MIRNA*/SPc complex at the mass ratio of 1:2 and 1:3. Representative complexes were enlarged. **Figure S3.** Enhanced delivery of SPc-loaded ds-*MIRNA* into protoplasts. The fluorescent photos were taken after 2 h incubation. **Figure S4.** QRT-PCR assay for target genes of *Arabidopsis*. The ds-*MIR166a*/SPc complex was applied to the root surface every 24 h, and the samples were collected on 6 days after the treatment. Triplicate biological replicates were used for each treatment. Different letters on columns indicate significant differences (Duncan’s multiple range test, *P* < 0.05). **Figure S5.** Photo of maize phenotype among various treatments. The ds-*MIR164b*/SPc complex was applied to the root surface every 24 h, and the photo was taken on 4 days after the treatment. Scale bar: 1 cm.

## Data Availability

The datasets used and/or analyzed during the current study are available from the corresponding author on reasonable request.
