# Supplementary material for: Construction and application of star polycation nanocarrier-based microRNA delivery system in Arabidopsis and maize
Source: J Nanobiotechnology. 2022 May 7;20:219. doi: 10.1186/s12951-022-01443-4 (PMC9077854; doi:10.1186/s12951-022-01443-4)
Supplement: Supplementary file 1 — Additional file 1: Table S1. The primers used in the current study. Figure S1. The pH test for ds-MIRNA/SPc complex on pH test strips. Different colors represent the corresponding pH values reference to the right color table. Figure S2. Representative TEM images of ds-MIRNA/SPc complex at the mass ratio of 1:2 and 1:3. Representative complexes were enlarged. Figure S3. Enhanced delivery of SPc-loaded ds-MIRNA into protoplasts. The fluorescent photos were taken after 2 h incubation. Figure S4. QRT-PCR assay for target genes of Arabidopsis. The ds-MIR166a/SPc complex was applied to the root surface every 24 h, and the samples were collected on 6 days after the treatment. Triplicate biological replicates were used for each treatment. Different letters on columns indicate significant differences (Duncan’s multiple range test, P < 0.05). Figure S5. Photo of maize phenotype among various treatments. The ds-MIR164b/SPc complex was applied to the root surface every 24 h, and the photo was taken on 4 days after the treatment. Scale bar: 1 cm. [file 12951_2022_1443_MOESM1_ESM.docx]

**Short Communication**

**Construction and application of star polycation nanocarrier-based microRNA delivery system in *Arabidopsis* and maize**

Jia Yang^1^**^†^**, Shuo Yan^2^**^†^**, Shipeng Xie^1^, Meizhen Yin^3^, Jie Shen^2^, Zhaohu Li^1^, Yuyi Zhou^1*^ and Liusheng Duan^1,4*^

* Correspondence: zhouyuyi@cau.edu.cn (Y. Z.) or duanlsh@cau.edu.cn (L. D.)

† Jia Yang and Shuo Yan have contributed equally to this work

^1^ State Key Laboratory of Plant Physiology and Biochemistry, Engineering Research Center of Plant Growth Regulator, Ministry of Education & College of Agronomy and Biotechnology, China Agricultural University, No. 2 Yuanmingyuan West Road, Haidian District, Beijing 100193, P.R. China

^2^ Department of Plant Biosecurity and MOA Key Laboratory for Monitoring and Green Management, China Agricultural University, No. 2 Yuanmingyuan West Road, Haidian District, Beijing 100193, P.R. China

^3^ State Key Laboratory of Chemical Resource Engineering, Beijing Laboratory of Biomedical Materials, Beijing University of Chemical Technology, No. 15 North Third Ring East Road, Chaoyang District, Beijing 100029, P. R. China

^4^ College of Plant Science and Technology, Beijing University of Agriculture, Beijing, 102206, P. R. China

**Table S1.** The primers used in the current study.

| **Gene** | **Primer** | **Primer sequence (5'-3')** |
| --- | --- | --- |
| **For ds-*MIRNA* synthesis** | | |
| *MIR166a* | F | GGATCCTAATACGACTCACTATAGGAGGGGCTTTCTCTTTTGAG |
|  | R | GGATCCTAATACGACTCACTATAGGAGGGAGCAACAATTGGGG |
| *MIR164b* | F | GGATCCTAATACGACTCACTATAGGTAGACGGTGGCTGTGCGT |
|  | R | GGATCCTAATACGACTCACTATAGGAAGACAGTTCGTGCTCGGT |
| **For miRNA first strand cDNA synthesis** | | |
| *miR166a* |  | GTCGTATCCAGTGCGTGTCGTGGAGTCGGCAATTGCACTGGATACGACGGGGAATG |
| *miR164b* |  | GTCGTATCCAGTGCGTGTCGTGGAGTCGGCAATTGCACTGGATACGACTGCACGTG |
| **For real-time PCR** | | |
| *miR166a* | F | GAGTCGGACCAGGCTTCAT |
| *miR164b* | F | GGGTGGAGAAGCAGGGCA |
| *General R* | R | CAGTGCGTGTCGTGGAGT |
| *CHC* | F | ATGACTTGGAAACTGCTGCG |
|  | R | GCACAACCAAGTTCTCAGCA |
| *SAL1* | F | CAAGGTCAAGAAGGCGATCG |
|  | R | CGATGGACTGCATGGACTTG |
| *Rab* | F | ATTCGATGTTGCAAGCAGGC |
|  | R | ATTTTACGGAACAGCGCCTT |
| *AP2* | F | CAATGGTCAAGGTGAGTGCC |
|  | R | TTGCTGCAGTCCAACATCAG |
| *EHD1* | F | ACGAGGAGTTTGCAAAGGTG |
|  | R | GCATTCACTCGTACGGGTTC |
| *ARF* | F | CCAAGCCCGAGAACAAGATG |
|  | R | CATCAGTCCATCCATGCTGC |
| *NAC1* | F | CAGCTCCACACCTGTACGT |
|  | R | CCATGCTCAGCGACTTGATG |
| *ZmUBC* | F | TGGTTGTGGCTTCGTTGGTT |
|  | R | GCTGCAGAAGAGTTTTGGGTACA |
| *PHV* | F | TGATTGGGATGAAGCCTGGT |
|  | R | CACATCGACAGTCACGGAAC |
| *PHB* | F | GGTCAGCAACATCAACAGCA |
|  | R | ACCAGGCTTCATCCCAATCA |
| *REV* | F | TGCGGTTAATGGGTTTGGTG |
|  | R | CCCACTCAGATCGATGCTCT |
| *ATHB8* | F | AAAACAGCGAAAAGAGGCGT |
|  | R | TGGTTTTGAGGATGTTGGCG |
| *ATHB15* | F | TTCGGTTTCTGAGGGAGCAT |
|  | R | ACGATTGCATCTTCAGGGGA |
| *AtActin-2* | F | ACGGTAACATTGTGCTCAGTGGTG |
|  | R | CTTGGAGATCCACATCTGCTGGA |


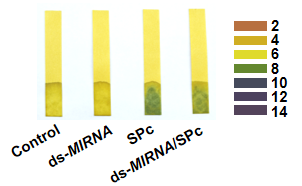


**Figure S1.** The pH test for ds-MIRNA/SPc complex on pH test strips. Different colors represent the corresponding pH values reference to the right color table.


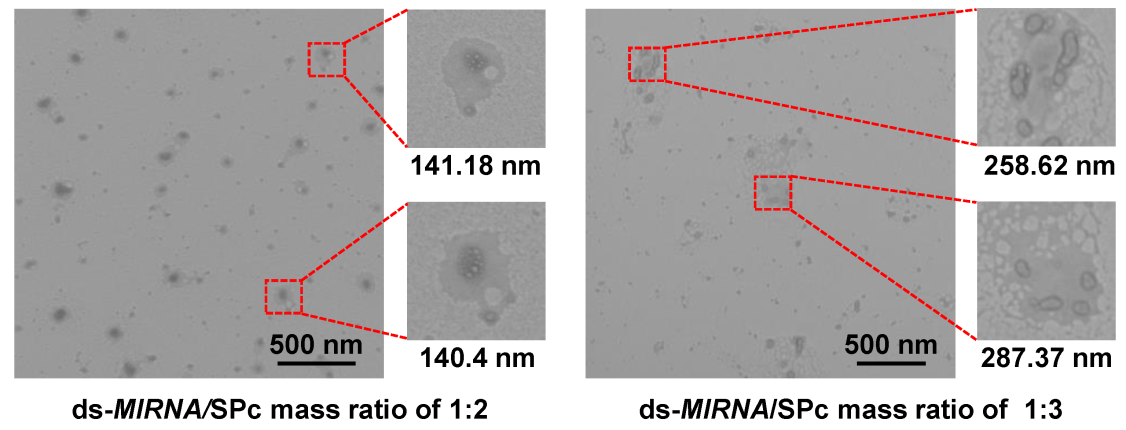


**Figure S2.** Representative TEM images of ds-*MIRNA*/SPc complex at the mass ratio of 1:2 and 1:3. Representative complexes were enlarged.


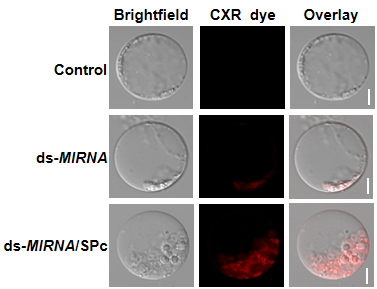


**Figure S3.** Enhanced delivery of SPc-loaded ds-*MIRNA* into protoplasts. The fluorescent photos were taken after 2 h incubation. Scale bar: 10 µm.


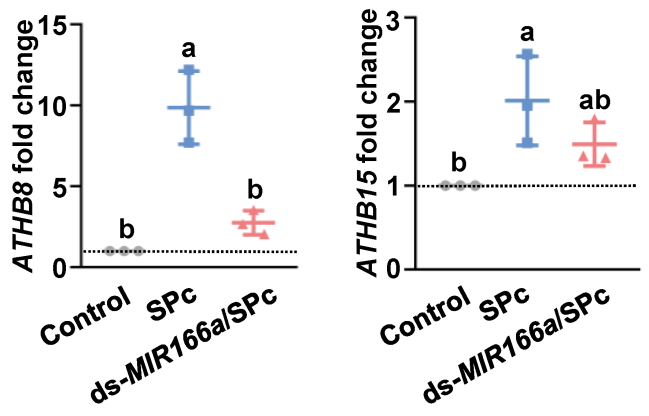


**Figure S4.** QRT-PCR assay for target genes of *Arabidopsis*. The ds-*MIR166a*/SPc complex was applied to the root surface every 24 h, and the samples were collected on 6 d after the treatment. Triplicate biological replicates were used for each treatment. Different letters on columns indicate significant differences (Duncan’s multiple range test, *P*<0.05).


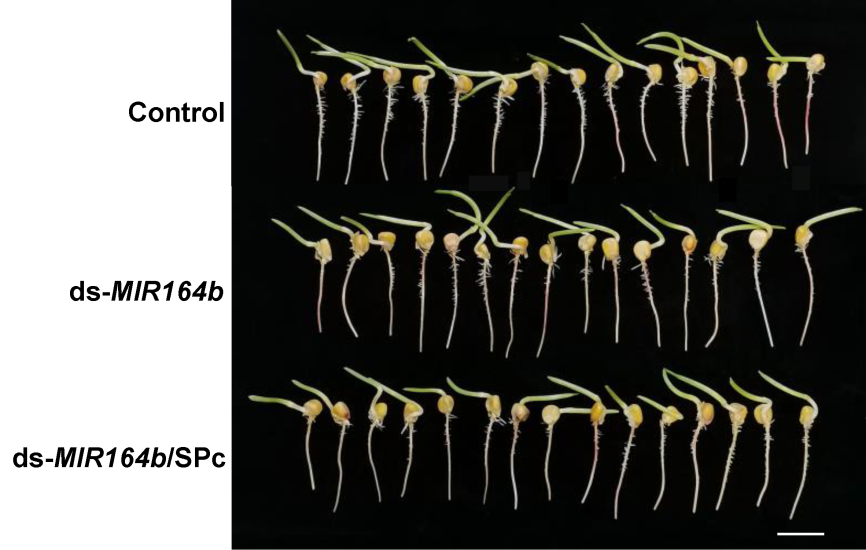


**Figure S5.** Photo of maize phenotype among various treatments. The ds-*MIR164b*/SPc complex was applied to the root surface every 24 h, and the photo was taken on 4 d after the treatment. Scale bar: 1 cm.
